# Pdgfrα-Cre mediated knockout of the aryl hydrocarbon receptor protects mice from high-fat diet induced obesity and hepatic steatosis

**DOI:** 10.1371/journal.pone.0236741

**Published:** 2020-07-30

**Authors:** Francoise A. Gourronc, Kathleen R. Markan, Katarina Kulhankova, Zhiyong Zhu, Ryan Sheehy, Dawn E. Quelle, Leonid V. Zingman, Zoya B. Kurago, James A. Ankrum, Aloysius J. Klingelhutz

**Affiliations:** 1 Department of Microbiology and Immunology, University of Iowa, Iowa City, IA, United States of America; 2 Department of Neuroscience and Pharmacology, Fraternal Order of Eagles Diabetes Research Center, University of Iowa, Iowa City, IA, United States of America; 3 Department of Pediatrics, University of Iowa, Iowa City, IA, United States of America; 4 Department of Internal Medicine, University of Iowa, Iowa City, IA, United States of America; 5 Department of Pharmacology, Kansas City University, Kansas City, KS, United States of America; 6 Department of Neuroscience and Pharmacology, Fraternal Order of Eagles Diabetes Research Center, University of Iowa, Iowa City, IA, United States of America; 7 Department of Internal Medicine, Fraternal Order of Eagles Diabetes Research Center, University of Iowa, Iowa City, IA, United States of America; 8 Department of Oral Biology and Diagnostic Sciences, Department of Pathology, Augusta University, Augusta, GA, United States of America; 9 Roy J. Carver Department of Biomedical Engineering, Fraternal Order of Eagles Diabetes Research Center, University of Iowa, Iowa City, IA, United States of America; 10 Department of Microbiology and Immunology, Fraternal Order of Eagles Diabetes Research Center, University of Iowa, Iowa City, IA, United States of America; The Ohio State University, UNITED STATES

## Abstract

Aryl hydrocarbon receptor (AHR) agonists such as dioxin have been associated with obesity and the development of diabetes. Whole-body Ahr knockout mice on high-fat diet (HFD) have been shown to resist obesity and hepatic steatosis. Tissue-specific knockout of Ahr in mature adipocytes via adiponectin-Cre exacerbates obesity while knockout in liver increases steatosis without having significant effects on obesity. Our previous studies demonstrated that treatment of subcutaneous preadipocytes with exogenous or endogenous AHR agonists disrupts maturation into functional adipocytes *in vitro*. Here, we used platelet-derived growth factor receptor alpha (Pdgfrα)-Cre mice, a Cre model previously established to knock out genes in preadipocyte lineages and other cell types, but not liver cells, to further define AHR’s role in obesity. We demonstrate that Pdgfrα-Cre Ahr-floxed (Ahr^fl/fl^) knockout mice are protected from HFD-induced obesity compared to non-knockout Ahr^fl/fl^ mice (control mice). The Pdgfrα-Cre Ahr^fl/fl^ knockout mice were also protected from increased adiposity, enlargement of adipocyte size, and liver steatosis while on the HFD compared to control mice. On a regular control diet, knockout and non-knockout mice showed no differences in weight gain, indicating the protective phenotype arises only when animals are challenged by a HFD. At the cellular level, cultured cells from brown adipose tissue (BAT) of Pdgfrα-Cre Ahr^fl/fl^ mice were more responsive than cells from controls to transcriptional activation of the thermogenic uncoupling protein 1 (Ucp1) gene by norepinephrine, suggesting an ability to burn more energy under certain conditions. Collectively, our results show that knockout of Ahr mediated by Pdgfrα-Cre is protective against diet-induced obesity and suggest a mechanism by which enhanced UCP1 activity within BAT might confer these effects.

## Introduction

Metabolic syndrome, a cluster of conditions (i.e. increased blood pressure, high blood sugar, central adiposity, elevated cholesterol or triglyceride levels) that increase the risk of heart disease, stroke, and type II diabetes [1], has increased dramatically in the past several decades in the U.S. and worldwide leading to enormous health-related costs [2, 3]. Adipose tissue is critical for normal metabolism and its dysfunction plays an essential role in the development of metabolic syndrome [4–6]. Adipose tissue is necessary for regulation of inflammation as well as secretion of adipokines such as adiponectin and leptin [7]. Adipose tissue is much more diverse than previously appreciated and brown, white, and beige adipose tissues play distinct roles in energy homeostasis. White adipose tissue (WAT) is found in different anatomical depots (e.g. subcutaneous and visceral) each with different attributes while BAT is found predominantly intrascapular in rodents and primarily within deep regions of the neck in humans [7–9]. Over-accumulation of triglycerides in mature white and brown adipocytes causes them to become hypertrophic, inflammatory, and pathological.

More than 10% of adipocytes in the human body are replaced annually through adipogenesis of precursor stem cells [10]. Adipogenesis provides flexibility to meet metabolic needs but also a vulnerability as endogenous and environmental factors (effectors or repressors) can disrupt normal adipogenesis [11]. Disruption of adipogenesis can result in stress on mature adipocytes leading to dysfunctional adipose tissue and disease [4, 10]. This dysfunction in adipogenesis and adipose tissue results in loss of insulin sensitivity in adipocytes, an increase in cytokine production, and loss of adipokine signaling [7]. Loss of insulin sensitivity and inhibition of the thermogenic response in adipose tissue are key initiating events in the development of metabolic syndrome [12].

The aryl hydrocarbon receptor (AHR), was first identified as the mediator of the toxin TCCD (2,3,7,8-Tetrachlorodibenzodioxin; also referred to as dioxin) [13]. AHR contains a promiscuous ligand-binding pocket that can bind to many types of endogenous and exogenous compounds [14]. Upon activation, AHR goes to the nucleus and binds a co-activator called ARNT (aryl hydrocarbon receptor nuclear translocator) to activate or repress numerous genes [15, 16]. AHR is expressed ubiquitously in fetal and adult tissues including adipose tissue [15].

AHR has been implicated in several physiologic and pathologic conditions including the development of metabolic syndrome [17–21]. Studies have linked dioxin exposure to an increased risk for diabetes [22] and other studies associate exposure to dioxin-like PCBs (polychlorinated biphenyls) with the development of insulin resistance and diabetes [23–27]. The mechanisms by which AHR ligands cause or exacerbate metabolic syndrome are unclear. Certain AHR agonists including dioxin have been shown to inhibit the proper maturation of precursor cells into adipocytes [28–32]. In published studies, we showed that PCB126 causes a proinflammatory response in preadipocytes and inhibits adipogenesis [33, 34]. In addition to man-made AHR ligands, several endogenous and microbiome-derived metabolites can act as AHR agonists [35]. These include kynurenine, FICZ, indole, and indoxyl sulfate (IS), all tryptophan metabolites.

Whole body Ahr knockout mice are known to exhibit developmental defects and have decreased fertility [36, 37]. Systemic Ahr deficiency in mice and rats has been shown to protect against high fat diet (HFD) induced obesity, hepatic steatosis, insulin resistance and inflammation [38–40]. Chemical inhibition of AHR has also protects against obesity caused by HFD [38, 41]. Conversely, mice with an Ahr allele that confers more sensitivity to AHR ligands were found to be more susceptible to HFD induced obesity [42]. What cells and tissues are directly involved in these AHR-mediated effects remains unclear.

Tissue specific models of Ahr loss have yielded differing results compared to whole body knockouts or chemical inhibition studies. For example, a recent study in which Ahr was ablated in a tissue-specific manner through expression of Cre from an adiponectin promoter (i.e. in mature adipocytes) caused an increase in obesity on HFD at baseline [43]. Our in vitro studies would suggest that preadipocytes, not adipocytes, are more susceptible to effects mediated by activated AHR [33]. Interestingly, liver-specific knockout of Ahr in mice had no effect on weight or adiposity in response to HFD but did lead to increased liver steatosis [39].

Clearly, questions remain as to how AHR mediates effects on obesity and steatosis when mice are on a HFD. To address this issue, we generated mice in which Ahr was selectively knocked out in cells that expressed Pdgfrα-Cre. This model has been used to inactivate genes in preadipocyte lineages, before they become adipocytes [44–50]. While this Cre model can also lead to Ahr knockout in certain tissues other than preadipocytes and adipocytes, it does not result in knockout in the liver [46], thus allowing an assessment of effects that are not directly related to the AHR’s well-known role in the liver [37, 51, 52]. We found that Pdgfrα-Cre mediated knockout of Ahr protected mice from HFD induced obesity and liver steatosis. Our results indicate that AHR activity in cell lineages that express Pdgfrα, which includes preadipocytes and adipocytes, is important for mediating the effects of HFD in mice.

## Materials and methods

### Animals

Ahr^flox/flox^ (Ahr^fl/fl^) C57/BL6 mice (Jackson Labs 006203) have been described previously [37]. The Pdgfrα-Cre mouse line (Jackson Labs 013148) has been used previously in preadipocyte lineage tracing studies and in studies to knockout genes in preadipocytes [44–50]. It should be noted that the Pdgfrα-Cre is highly active in, but not limited to, preadipocytes of adipose lineages [46]. A lineage tracing study demonstrated less than 5% recombination of cells in liver tissue [44]. Both strains of mice were purchased from Jackson Laboratories. The Ahr^fl/fl^ were obtained as a homozygous breeding pair. To achieve tissue-specific knockout of Ahr, Pdgfrα-Cre^pos/neg^ (Cre always maintained in heterozygous state and only in males) mice were bred to homozygosity for floxed Ahr (Ahr^fl/fl^). For generation of Pdgfrα-Cre^pos^ Ahr knockout mice and controls, male Pdgfrα-Cre^pos/neg^ (heterozygous Cre)/Ahr^fl/fl^ were bred to female Ahr^fl/fl^ mice that did not express Cre. The offspring showed a 50:50 ratio of Cre^pos^ and Cre^neg^ genotypes as assessed by PCR for Cre. Male mice were used in this study because male C57/BL6 mice exhibit significant and consistent development of obesity and insulin resistance when on HFD [53].

To verify Ahr recombination in adipose tissue, BAT, subcutaneous and visceral WAT, muscle, heart, liver, kidney and spleen tissues were removed from Pdgfrα-Cre^pos^ adult mice and processed for DNA isolation after homogenization. Verification of recombination in different tissues or lack thereof was performed using published primers and conditions [37]. An explanation of expected patterns of recombination (excised) or non-recombination (unexcised) of the PCR products is shown in S1A Fig.

Mice on HFD were fed 60% high fat diet (HFD; Research Diets, D12492i) for the indicated time. Control diet (Research Diets, D12550j) with 10% fat and a matched calorie content (provided by complex carbohydrates) was used for comparison. Mice were placed on their specific diets starting at 6–7 weeks of age. Different genotypes were dispersed randomly in cages. The number of mice used in each experiment is indicated in the figure legends. Mouse weights were measured weekly.

This study was carried out in strict accordance with the recommendations in the Guide for the Care and Use of Laboratory Animals of the National Institutes of Health. The protocol was approved by the University of Iowa IACUC (Protocol number 8091538). Tail snips were only taken at the time of weaning at 3 weeks of age. Mice were monitored daily by the Animal Care Facility staff for signs of distress and/or fighting. Fighting animals were separated. All efforts were used to minimize suffering during procedures. Adult animals were euthanized using CO_2_ followed by cervical dislocation to ensure death. Neonates were euthanized by rapid decapitation with a scissors.

### Glucose and insulin tolerance tests

Following a 6-hour fast, time 0 blood was collected via tail bleed followed by an intraperitoneal (i.p.) injection of glucose (2 g/kg for control diet and 1.3 g/kg for HFD). Different amounts of glucose were used for the control-fed and HFD groups because HFD-fed animals have a lower percent lean body mass as compared to total body weight thus potentially biasing results towards showing impaired glucose tolerance in the high-fat group [54–56]. Regardless, statistical comparisons were only made between mice that received the same amount of glucose per body weight. Tail blood was then collected into 300K2E microvette EDTA tubes (Sarstedt) over the course of 120 min and then centrifuged at 3000 rpm for 30 min at 4°C for the separation of plasma. Plasma glucose was then measured using the Autokit Glucose Reagent (WAKO) per manufacturer's instructions. For insulin tolerance tests (ITTs), mice were fasted 6 h. Time 0 blood was obtained via tail bleed followed by an intraperitoneal (i.p.) injection of insulin (at 0.75 units/kg). Tail blood was collected and plasma glucose analyzed as described above.

### Fat and liver tissue histology

After euthanizing, mice were dissected to remove liver and fat depots (subcutaneous, visceral, and brown). Tissues were fixed in 10% phosphate-buffered formalin and then processed, sectioned and stained by hematoxylin and eosin (H&E) using standard pathology methods at the University of Iowa Comparative Pathology Core. Coded liver sections were evaluated and scored by a pathologist blinded to the experimental conditions. Each of the sections was analyzed by photographing 10 non-overlapping high-power fields (400x) centered on the portal vein for consistency, and the percentage of lipid-containing hepatocytes was recorded, followed by statistical analysis of the scores (GraphPad Prism). Adipocyte number and size in inguinal WAT (iWAT) or epididymal WAT (eWAT), also referred to as subcutaneous and visceral WAT, respectively, were quantified using Adiposoft software (ImageJ) [57]. Because of small size and cellular complexity, BAT was not amendable to this type of analysis.

### Adiposity measurement

Body composition was measured using a rodent-sized NMR machine (Bruker Minispec LF50) at the Fraternal Order of Eagles Diabetes Research Center (FOEDRC) Metabolic Core. The percent lean or fat mass is calculated by dividing the lean or fat mass by the total weight and multiplying by 100. The addition of the percent lean and percent fat does not add up to one hundred percent because of additional mass from fluid and bone density not accounted for via NMR.

### Isolation and culturing of BAT from pups, NE-treatment, and Q-RT-PCR

Neonate mice were euthanized and dissected to isolate BAT. BAT from individual pups was dissociated using collagenase and cultured in one well of a 12-well plate according to published protocols [56]. Confluent wells were passaged 1:4 into 4 new wells. One well was used for isolation of DNA for genotyping for assessing the status of Cre and Ahr excision status (see above) whereas the other wells were used for norepinephrine (NE) treatments to induce a thermogenic response. Cells were treated with 10 μM NE or vehicle for 6 hours followed by RNA isolation. RNA was isolated using Trizol followed by column purification (RNA Easy, Qiagen) with DNase treatment. RNA was reverse transcribed according to published protocols [58]. Quantitative PCR was performed using primers for 18S (internal control) or Ucp1. Sequences of primers were Ucp1 forward, CAA GAG GAA GGG ACG CTC AC; Ucp1 reverse AGT TGT CGG GTT CAC CAT CC; Adiponectin forward GCA GAG ATG GCA CTC CTG GA; Adiponectin reverse CCC TTC AGC TCC TGT CAT TCC; 18S forward AGG GGA GAG CGG GTA AGA GA; 18S reverse GGA CAG GAC TAG GCG GAA CA.

### Statistical analysis

Statistical analysis was performed using GraphPad Prism software. Numbers of replicates/animals and the various tests that were performed are noted in the figure legends.

## Results

### Pdgfrα-Cre Ahr knockout mice are resistant to high-fat diet induced obesity and increased fat mass

We used a previously described Pdgfrα-Cre system that has been shown in lineage tracing studies to be active in preadipocyte lineages in order to test the function of the AHR in preadipocytes [44, 46]. This model has been used in different studies to knockout genes in preadipocytes and subsequently in the adipocytes that are derived from them [47–50]. For Ahr knockout, we bred Pdgfrα-Cre with Ahr^fl/fl^ on a pure C57/BL6 background. Mature Pdgfrα-Cre^pos/neg^/Ahr^fl/fl^ mice were then assessed for recombination in fat depots and other tissues by isolation of tissue and assessment of recombination by PCR as previously described [37]. High levels of recombination were detected in all fat depots, indicating that Pdgfrα-Cre caused excision of the Ahr floxed gene (S1A and S1B Fig). In contrast, minimal levels of recombination were observed in liver, as reported previously in a lineage tracing study [44]. Other tissues, including heart, spleen, and to some extent, kidney and muscle also exhibited recombination, indicating a low level of Ahr excision in these tissues, likely due to PDGFRα being expressed in certain cellular components (e.g. endothelial cells) of these tissues [59]. To verify clean knockout in preadipocytes, stromal vascular fractions were isolated and cultured from the BAT of pups that were either Pdgfrα-Cre^pos^ or Cre^neg^. The vast majority of the cells that grow from SVF are preadipocytes and, accordingly, PCR results indicated clear excision of Ahr in Pdgfrα-Cre^pos^ cells but not in Cre^neg^ cells (S1C Fig). Thus, as previously reported, the Pdgfrα-Cre is highly active in, but not limited to, preadipocytes of adipose lineages [46]. The low level of excision in the liver makes it a useful model for separating out those phenotypic changes following deletion of the AHR that are not directly associated with liver. No noticeable differences in the number of pups with the different Cre genotypes were observed. In an assessment of 8 breeding pairs, there were 17 male Pdfrgα-Cre^pos^ offspring and 18 male Cre^neg^ offspring or 48.6% and 51.4%, respectively, indicating that Pdgfrα-Cre knockout of Ahr does not significantly affect male survival.

**Fig 1:**
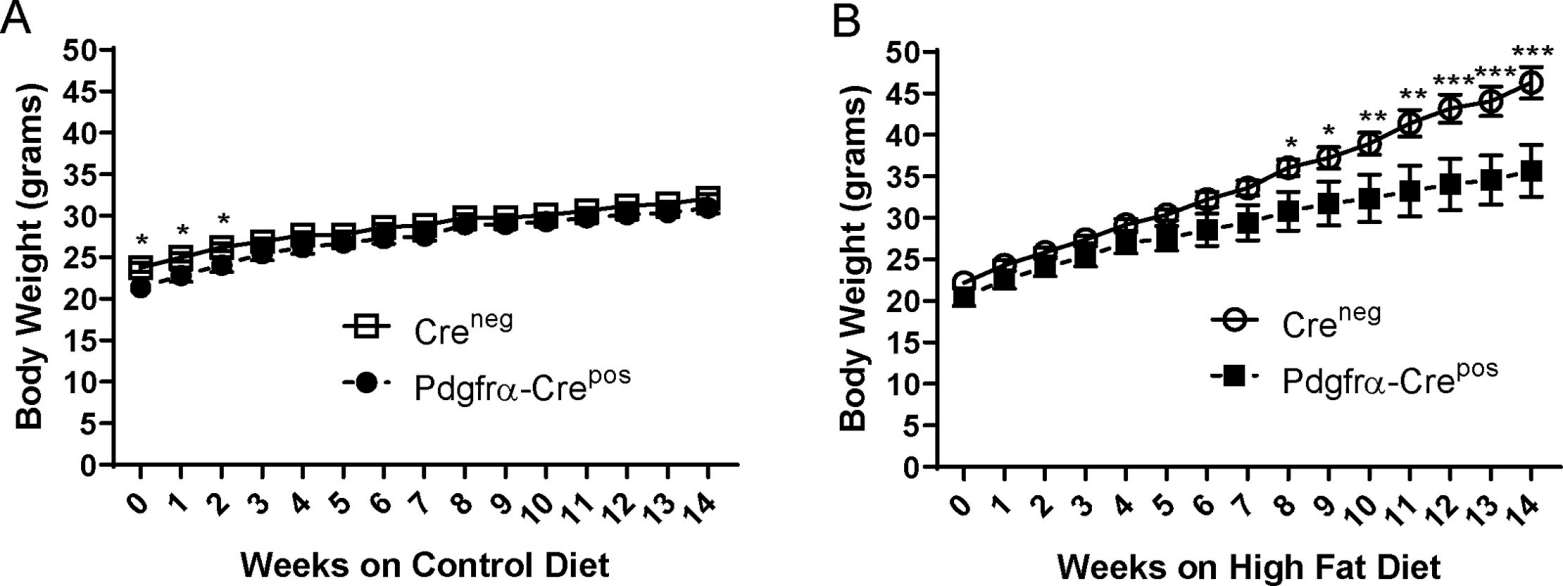
Effects of HFD (60% fat) or lower-fat (10% fat) calorie-matched control diet on weights of mice without Ahr knockout (Cre^neg^) or with Pdgfrα-Cre knockout (Pdgfrα-Cre^pos^). A. Weights of mice on control diet; B. Weights of mice on HFD. Mice were placed on HFD or control diet at 6 to 7 weeks of age and weighed weekly as described in the Materials and Methods. Cre^neg^ and Pdgfrα-Cre^pos^ groups consisted of 7 mice each for HFD and 6 mice each for control diet. Statistics were performed using 2-way ANOVA with multiple comparisons in GraphPad Prism. * <0.05, **<0.01, ***<0.001. Error bars represent standard error of the mean.

After weaning at 3 weeks, mice were kept on regular chow until 6–7 weeks of age during which time genotyping was performed for Cre expression. Pdgfrα-Cre^pos/neg^/Ahr^fl/fl^ knockout mice (referred to here and in figures as Pdgfrα-Cre^pos^) at 6–7 weeks of age were found to be more variable and, on average, statistically weighed less than Cre^neg^/Ahr^fl/fl^ wildtype controls (referred to here and in figures as Cre^neg^) (S2 Fig). The mice were randomly separated into HFD (60% fat) and calorie-matched control diet (10% fat) cages. While body weights of the Cre^neg^ and Pdgfrα-Cre^pos^ genotypes converged by week 3 on the control diet and remained similar for the rest of the experiment (Fig 1A), body weight between genotypes began to diverge at week 5 of HFD at which point the Cre^neg^ mice trended toward heavier weights, becoming significantly different by week 8 (Fig 1B). By the end of the observation period at 14 weeks, Cre^neg^ mice were, on average, more than 10 grams heavier than Pdgfrα-Cre^pos^ animals lacking Ahr in preadipocytes (Fig 1B). Of note, there was no significant difference in food intake between the genotypes on either HFD or control diet (S3 Fig). These results indicate the difference in body weights between genotypes was driven mainly by the HFD.

**Fig 2:**
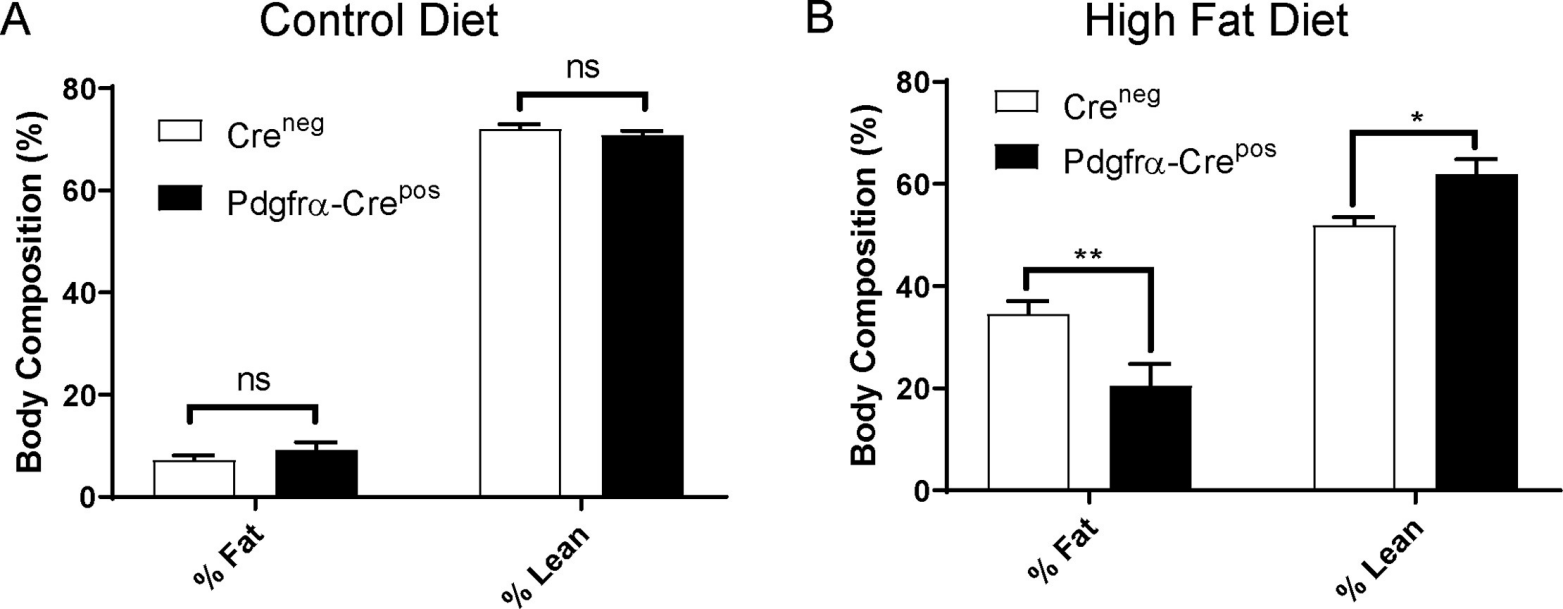
Effects of HFD or control diet on adiposity of mice without Ahr knockout (Cre^neg^) or with Pdgfrα-Cre knockout (Pdgfrα-Cre^pos^). A. Adiposity of mice on control diet; B. Adiposity of mice on HFD. Fat mass was assessed using NMR at 14 weeks on HFD or control in mice as described in the Materials and Methods. Cre^neg^ and Pdgfrα-Cre^pos^ groups consisted of 7 mice each for HFD and 6 mice each for control diet. The percent lean or fat mass is calculated by dividing the lean or fat mass by the total weight and multiplying by 100. The addition of the percent lean and percent fat does not add up to one hundred percent because of additional mass from fluid and bone density. Statistics were performed using a 2-way ANOVA with multiple comparisons in GraphPad Prism. * <0.05, **<0.01. Error bars represent standard error of the mean.

To assess body composition, both cohorts of mice on control diet or HFD were subjected to whole-body NMR at week 14. No differences in adiposity were observed between Cre^neg^ and Pdgfrα-Cre^pos^ genotypes on control diet (Fig 2A). In contrast, Pdgfrα-Cre^pos^ mice on HFD had higher lean mass and lower percent fat overall than Cre^neg^ mice (Fig 2B), indicating that Pdgfrα-Cre mediated knockout of Ahr protects against accumulation of fat.

### Glucose and insulin tolerance in Pdgfrα-Cre Ahr knockout mice

**Fig 3:**
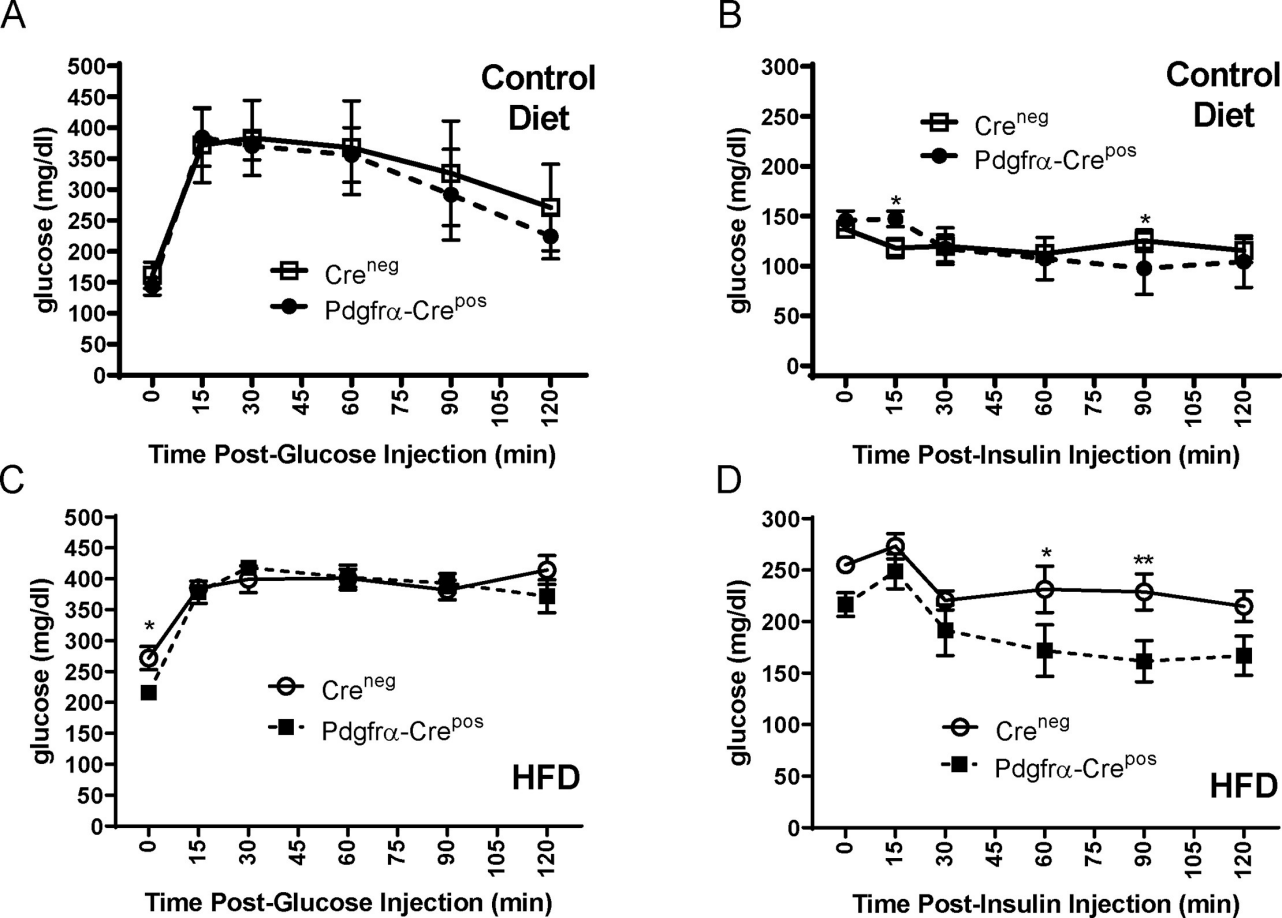
Effects of HFD or control diet on glucose and insulin tolerance in mice without AHR knockout (Cre^neg^) or with Pdgfrα-Cre Ahr knockout (Pdgfrα-Cre^pos^). Glucose tolerance test (GTT) (A) and insulin tolerance test (ITT) (B) on mice on control diet. GTT (C) and ITT (D) on mice on HFD. GTT and ITT were performed at 14 weeks on indicated diets as described in the Materials and Methods. Cre^neg^ and Pdgfrα-Cre^pos^ groups consisted of 7 mice each for HFD and 6 mice each for control diet. Error bars represent standard error of the mean. Asterisks above the curve represent statistical significance of comparisons of individual time points using 2-way ANOVA with multiple comparisons without corrections using Fishers LSD in GraphPad Prism. * <0.05, **<0.01. Analysis of AUC of overall glucose levels comparing different genotypes was also performed but no statistically significant differences were observed in any of the above comparison.

Since obesity is associated with the development of type II diabetes, we performed glucose tolerance tests (GTT) and insulin tolerance tests (ITT) on the mice on HFD or control diet. As seen in Fig 3A and 3B, there were no differences between Cre^neg^ and Pdgfrα-Cre^pos^ genotypes during a GTT or ITT while on the control diet. As expected for mice on long-term HFD, higher levels of basal glucose were observed in the HFD groups but, interestingly, there were also no differences between genotypes when on HFD in the ability to clear plasma glucose as measured by GTT (Fig 3C). HFD Pdgfrα-Cre^pos^ Ahr mice, however, displayed significantly enhanced insulin sensitivity indicated by ITT results at the individual 60- and 90-minute time points compared to HFD Cre^neg^ mice (Fig 3D). However, comparison of overall glucose levels at all time points using area under the curve (AUC) analysis between the Cre^neg^ and Pdgfrα-Cre^pos^ genotypes on HFD did not demonstrate statistically significant differences in the ITT (p = 0.07). Thus, although Pdgfrα-Cre mediated knockout of Ahr appeared to offer some protection against HFD-induced insulin resistance at certain time points, the overall effect on insulin sensitivity was not considered significant.

### Decreased hepatic steatosis and smaller adipocyte size in Pdgfrα-Cre Ahr knockout mice on HFD

**Fig 4:**
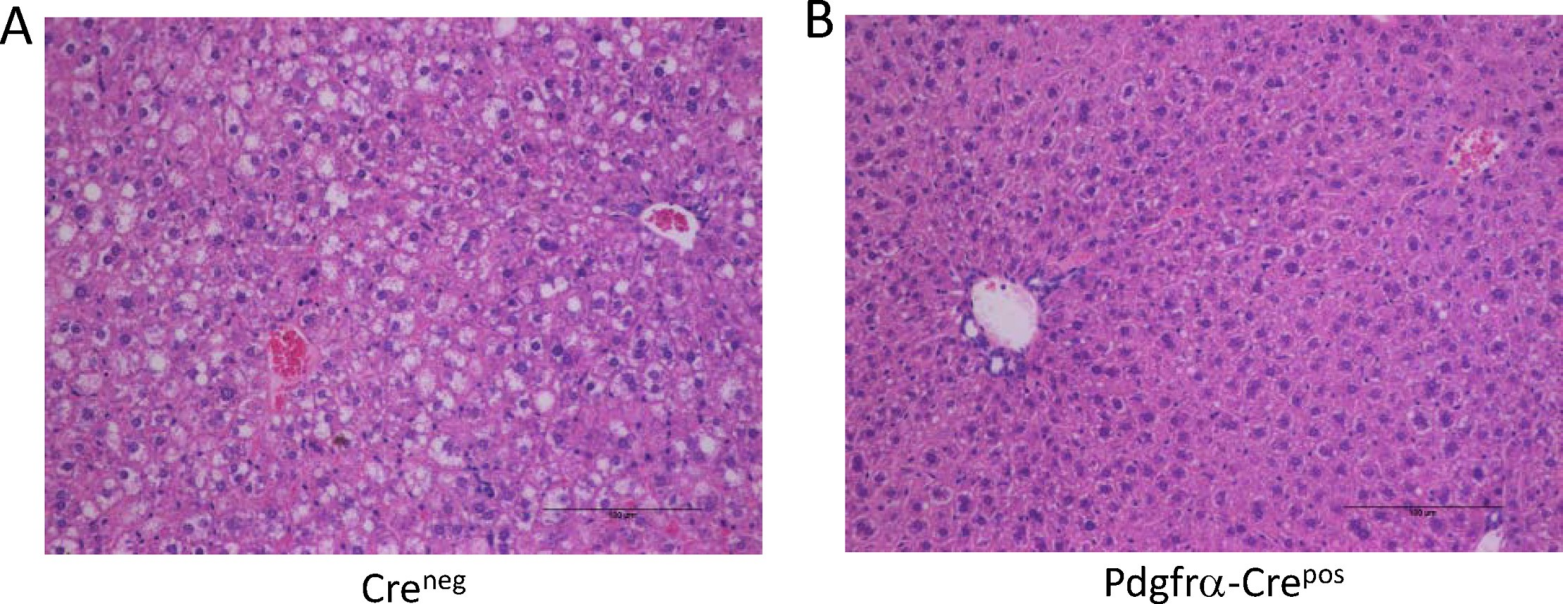
Liver pathology in mice on HFD without Ahr knockout (Cre^neg^) or with Pdgfrα-Cre knockout (Pdgfrα-Cre^pos^). Shown are representative examples from each genotype. A. Cre^neg^ on HFD, B. Pdgfrα-Cre^pos^ on HFD. Images were taken using a Nikon Eclipse E800 microscope at 200X. Scale bars are 100 micrometers.

Obesity is highly associated with liver steatosis (i.e. accumulation of lipid in hepatocytes) [60]. Therefore, livers obtained from mice on HFD were evaluated as described in the Materials and Methods. Examples of H&E sections Cre^neg^ and Pdgfrα-Cre^pos^ mice are shown in Fig 4. As expected, Cre^neg^ mice on HFD exhibited steatosis, demonstrated by numerous lipid vacuoles in the hepatocytes (Fig 4A, ranging from 15–20% in one of the samples, to over 75% in others (see S1 Table). Interestingly, Pdgfrα-Cre^pos^ Ahr knockout mice on HFD exhibited no steatosis, with less than 5% of hepatocytes containing lipid vacuoles in all samples examined (Fig 4B). The severity of steatosis was scored and ranked (S1 Table), demonstrating significant differences between Cre^neg^ and Pdgfrα-Cre^pos^ mice on HFD (p<0.05, Wilcoxon Rank-Sum). This result is of interest since Pdgfrα-Cre does not knock out Ahr in liver, indicating that protection against steatosis in the liver is likely due to the genetic deletion of Ahr in other tissues such as adipose.

**Fig 5:**
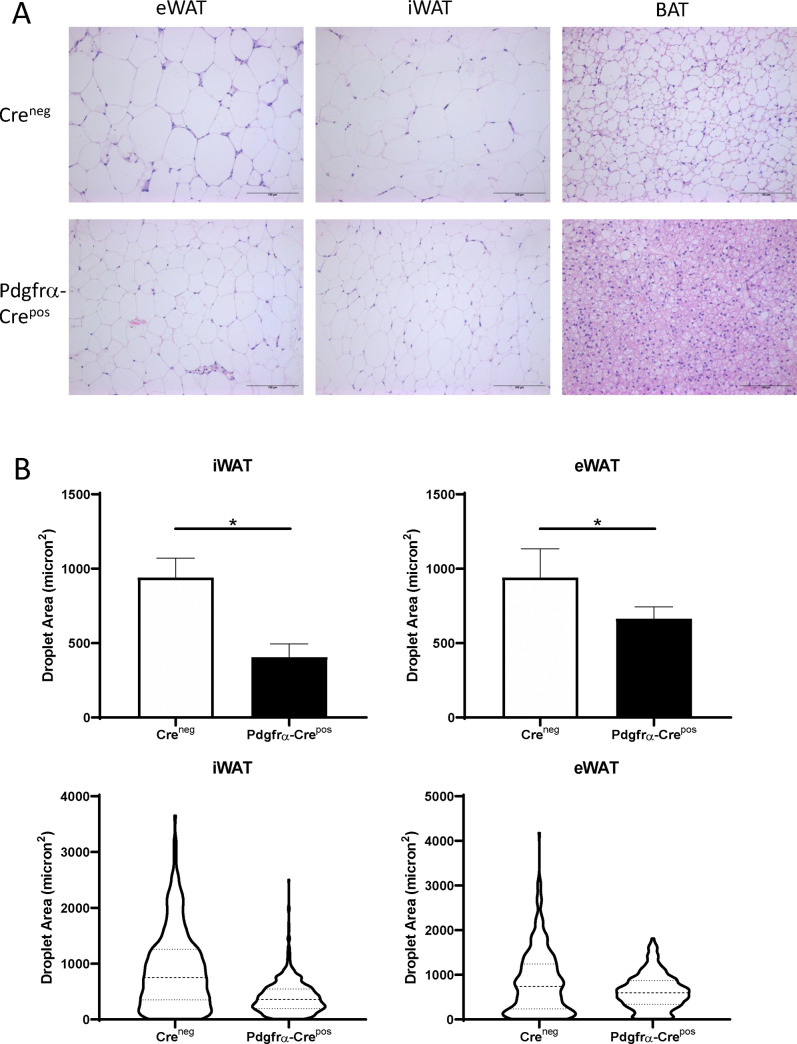
Adipose tissue pathology in mice on HFD without Ahr knockout (Cre^neg^) or with Pdgfrα-Cre Ahr knockout (Pdgfrα-Cre^pos^). A. Shown are representative examples of Cre^neg^ and Pdgfrα-Cre^pos^ mice on HFD for eWAT, iWAT, and BAT. Scale bars are 100 micrometers. B. Quantitation of adipocyte sizes in eWAT and iWAT fat depots of Cre^neg^ or Pdgfrα-Cre^pos^ mice on HFD. Quantitation was performed using the Adiposoft program in ImageJ. The upper panels represent the mean and the error bars are standard deviation. Unpaired T-test, *p<0.05. The lower panels contain descriptive graphs to show the 25^th^, 50^th^, and 75^th^ quartiles to illustrate the large differences ins droplet sizes between the groups.

Microscopic examination of H&E stained sections of various fat depots demonstrated that Pdgfrα-Cre^pos^ Ahr knockout mice on HFD had much small adipocytes than those in Cre^neg^ mice on HFD (Fig 5A). This was true of iWAT, eWAT, and BAT. Adiposoft software, a program developed specifically for characterization of WAT [57] was used to quantify adipocyte size in WAT. This analysis indicated that the iWAT and eWAT of Pdgfrα-Cre^pos^ Ahr knockout mice had on average smaller adipocytes with the distribution of adipocyte size narrower compared to controls (Fig 5B). Thus, our results suggest that Pdgfrα-Cre mediated knockout of Ahr protects against adipocyte hypertrophy in all fat depots of mice on HFD.

### BAT from Pdgfrα-Cre Ahr knockout mice exhibits greater thermogenic potential following norepinephrine treatment

**Fig 6:**
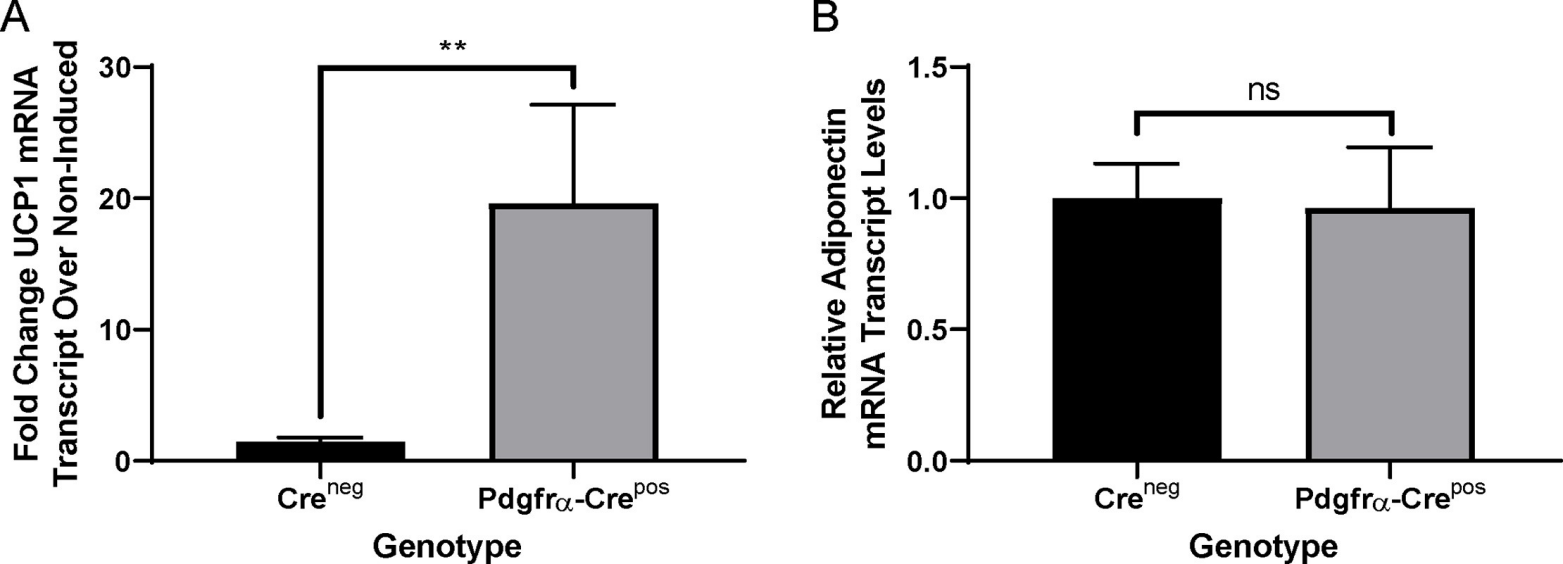
Norepinephrine induced induction of Ucp1 in BAT. A. Ratios of norepinephrine-induced Ucp1 in BAT from neonate pups of Cre^neg^ (no Ahr knockout) or Pdgfrα-Cre^pos^ Ahr knockout genotypes. Treatments and Q-RT-PCR were performed as described in the Materials and Methods comparing the ratio of Ucp1 transcript in NE-treated versus baseline in 4 Cre^neg^ and 6 Pdgfrα-Cre^pos^ littermates. B. Transcript levels of the differentiation marker, adiponectin, in cells of mice of different genotypes. Q-RT-PCR was performed as described in the Materials and Methods. Statistics were performed using a Wilcoxon nonparametric test in GraphPad Prism. Error bars represent standard error of the mean. **p<0.01.

Previously, it was shown that whole-body Ahr knockout mice on HFD have increased transcript levels of the thermogenic uncoupling gene Ucp1 in BAT as compared wild type mice, potentially explaining how Ahr knockout could protect against HFD induced obesity [38]. Thermogenic induction of Ucp1 is mediated through cold exposure or by beta adrenergic receptor activators such as norepinephrine [61]. To characterize the effects of Pdgfrα-Cre-mediated knockout of Ahr on BAT responses to thermogenic inducing agents, we isolated and cultured SVF from BAT of neonates, verified Ahr gene excision status (S1C Fig), differentiated the cells *in vitro*, and then stimulated the cultures with norepinephrine (NE). RNA was isolated to assess Ucp1 transcript levels by Q-RT-PCR. As shown in Fig 6A, the levels of NE-induced Ucp1 transcripts were significantly higher in Pdgfrα-Cre^pos^ Ahr knockout adipocytes than in Cre^neg^ adipocytes, indicating that knockout of Ahr leads to a more robust thermogenic response in BAT. Assessment of transcript levels of adiponectin, a marker of adipocyte differentiation, indicated no differences in ability of the cells from both genotypes to differentiate (Fig 6B). These results, combined with the smaller size of KO brown adipocytes and decreased brown adipocyte lipid accumulation, suggests increased level of energy expenditure in Pdgfrα-Cre Ahr knockout mice, presenting a plausible mechanism by which Pdgfrα-Cre mediated knockout protects against obesity and steatosis.

## Discussion

AHR continues to be a source of significant interest regarding its role in the development of metabolic syndrome, obesity, steatosis, cardiovascular disease, and diabetes. AHR responds to many different endogenous, bacterially-produced, and synthetic man-made compounds that include numerous persistent organic pollutants that people are exposed to on a regular basis [13, 14]. Further, there is compelling evidence that AHR activation causes inflammation in endothelium, the liver, and adipose tissue [32, 34, 62–64]. Depending on the context and the type of AHR ligand, AHR has been shown to inhibit adipogenesis or act as an obesogen. Our previous studies using human cells indicated that the AHR agonist, PCB126, acts through AHR on preadipocytes to inhibit adipogenesis [28–32] and blocks norepinephrine-mediate induction of UCP1 in adipocytes derived from PCB126-treated preadipocytes [65]. Interestingly, in the current study, we found that knock out of Ahr using Pdgfrα-Cre, a model that has been shown in lineage tracing studies to act in preadipocytes, but not the liver, protected mice from HFD-induced obesity and steatosis. Adipocytes derived from preadipocytes of BAT from Pdgfrα-Cre Ahr knockout mice were more responsive to norepinephrine-mediated induction of the thermogenic uncoupling protein UCP1. Our results suggest that the effects of AHR on metabolic health are at least partly mediated through adipose tissue and that effects on UCP1 induction may play a role.

Other groups, using whole-body Ahr knockout, have also demonstrated a protective effect of AHR loss against HFD-induced obesity [38, 66, 67]. In those studies, protection against HFD-induced insulin resistance was also observed. While the results of our studies with the Pdgfrα-Cre mediated knockout mice were suggestive of protection against insulin resistance, the effects were not overtly significant. This could be because Ahr knockout in other tissues besides those that express Pdgfrα are important for protection against insulin resistance. Regardless, the collective results of these studies point to the possibility that HFD acts through AHR to cause obesity and steatosis, potentially through alteration in production of a metabolite (or metabolites) that acts as an AHR ligand. While the identity of this metabolite is unknown, there is evidence that HFD can alter levels of known AHR agonists such as tryptophan catabolites generated in the kynurenine pathway, for example [68]. Given that knockout of AHR (whole body or Pdgfrα-Cre mediated) can protect against obesity, it is reasonable to propose that this metabolite is an AHR agonist. If it were, instead, an antagonist then it would follow that Ahr knockout would more likely increase obesity, which we did not observe. One possibility is that the metabolite or metabolites could act on preadipocytes and/or adipocytes to increase adipogenesis thereby increasing overall lipid accumulation in adipocytes to cause obesity. Another possibility, and one that is favored by our current data, is that an HFD-induced AHR agonist blocks preadipocytes from becoming thermogenically responsive adipocytes, thus decreasing energy expenditure and contributing to obesity. Our data would suggest that this could be at the level Beta-adrenergic receptor and/or the level of Ucp1 transcription Knockout of Ahr in BAT preadipocytes/adipocytes may prevent these AHR agonist-mediated effects. Similarly, HFD-induced AHR agonists may also prevent beiging in subcutaneous adipocyte lineages, inhibiting thermogenic responses in these fat depots, as well. In future studies, it will be of interest to assess whole body energy expenditure in the Pdgfrα-Cre Ahr knockout mice compared to controls, particularly in the context of known AHR agonists, to determine if there are differences.

Interestingly, a role for AHR in regulation of energy expenditure through its interaction with circadian clock proteins has been explored previously [38, 67, 69, 70]. AHR forms a heterodimer with the circadian clock protein Bmal1 and functionally inhibits CLOCK/BMAL1 activity. Physiological activation of AHR through naturally occurring endogenous ligands may inhibit clock function. Whole-body knock out of Ahr was shown to be associated with higher levels of Ucp1 transcript levels as compared to controls in brown fat of mice on HFD [38]. This same study also reported that whole body Ahr knockout enhances behavioral responses to changes in light-dark cycle and increased the rhythmic amplitude of circadian clock genes as well as altered rhythms of glucose and insulin [67]. These studies demonstrate an already established role for the AHR in regulating energy metabolism, thermogenic responsiveness and glucose and insulin homeostasis.

Initially, our results along with studies published by others using whole body Ahr knockout appear in conflict with a report in which it is was shown that knockout of Ahr in mature adipocytes through expression of adiponectin-Cre actually exacerbated HFD-induced obesity [33]. One way to explain this discrepancy is that knockout in preadipocytes or whole-body knockout would be expected to have more profound effects on how HFD affects the process of adipogenesis and the maturation of cells into functional thermogenically-responsive adipocytes. Delay of knockout of Ahr until the adipogenesis program is fully activated (when adiponectin is expressed) may result in a completely different phenotype with different responses to AHR ligands. These converse effects also suggest cell autonomous versus non-autonomous actions of the AHR at the level of adipose tissue. Thus, the timing of Ahr knockout during the course of adipogenesis may be important for determining the outcome.

We cannot rule out other cell types such as skeletal muscle and heart may play a role in mediating the effects of the AHR that we observed in our studies given that Pdgfrα-Cre is active in other cell types. Muscle is highly relevant to energy expenditure. While others have reported that Pdgfrα-Cre activity is low in muscle [44] and our results indicate minimal Ahr excision in muscle, it is still possible that Ahr knockout in certain muscle cells are playing a role in the phenotype that we observed. Clearly, further studies are warranted to identify the both the potential AHR metabolite and the target cell population(s).

Confounding the interpretation of the role of AHR in metabolic syndrome or any other diseases in future studies is the observation that not all AHR agonists act in the same fashion. Depending on the AHR agonists, different effects on obesity in animals have been observed. It has been shown, for example, that PCB77, a dioxin-like PCB that activates AHR, caused obesity in mice [71]. A similar finding was observed for dioxin [72]. In contrast, the *P*. *aeruginosa* pigment molecule, pyocyanin, also an AHR activator, caused inhibition of adipogenesis resulting in wasting syndrome [73]. A recent study identified indigo, a naturally occurring AHR ligand, as having anti-inflammatory properties in visceral adipose tissue that effectively protected against HFD-induced glucose intolerance [74]. In another study it was shown that people and animals with metabolic syndrome had reduced levels of AHR agonist activity in fecal samples [75]. In this case, the deficiency was attributed to the gut microbiota, and supplementation with AHR agonist or a *Lactobacillus* strain with high AHR ligand-production capacity improved dietary and genetic induced metabolic impairments. Thus, certain AHR agonists may be detrimental in causing metabolic syndrome and others might be protective. Further, some compounds that act as AHR agonists in one context or concentration may act as AHR antagonists in others. Finally, certain AHR agonists and antagonists may act very differently on rodent versus human cells.

In summary, our studies demonstrate a significant role for AHR in obesity and steatosis in male mice on a high-fat diet. The Pdgfrα-Cre specific knockout of Ahr supports a role for AHR that does not directly involve the liver but may be mediated in part through effects on preadipocytes/adipocytes. Further studies, using additional tissue-specific and inducible knockout models, as well as determination of what AHR agonists are produced by high-fat diet will help to define how modulating AHR activities may be useful in prevention and therapeutic interventions for obesity and diabetes.

## Supporting information

S1 Fig Cre-mediated recombination of Ahr in different tissues and preadipocytes. A. Pdgfrα-Cre expression causes recombination to occur between the two loxP sites (black diamonds) surrounding exon 2 of Ahr (referred to as floxed), excising the exon and leaving one remaining loxP site. PCR using three primers (P1, P2, and P3) was performed to determine recombination (excision) status. In cells where no recombination occurs (upper structure), P2 and P3 amplify a 140 bp fragment (P1 and P3 are too far apart to achieve any appreciable amplification). In cells where recombination and excision occur (lower structure), the P2 site is removed and P1 and P3 are brought in close proximity to allow amplification of a 180 bp band. B. To verify AHR recombination, the indicated tissues were removed from Pdgfrα-Cre^pos^ Ahr^fl/fl^ adult mice and processed for DNA. PCR was performed by using published primers [37]. The arrow indicates the upper 180 bp band that demonstrates recombination of the floxed Ahr gene (excised) in the tissue. The lower 140 bp band represents non-recombined (unexcised) Ahr. The pattern is typical of complex tissue such as adipose tissue where not all the different cell types express Pdgfrα-Cre. **C**. Stromal vascular fraction (SVF) consisting mainly of preadipocytes from BAT of 10 neonatal pups derived from the breeding of male Pdgfrα-Cre^pos/neg^ (heterozygous Cre)/Ahr^fl/fl^ and female Ahr^fl/fl^ mice that did not express Cre was isolated and cultured for one passage before DNA extraction and assessment for Cre positivity and Ahr recombination (excision) by PCR as described in A and in the Materials and Methods. Only the SVF that was Cre positive exhibited a pattern that verified excision.
(PDF)

S2 Fig Starting weights of 6- to 7-week old male Creneg or Pdgfrα-Crepos Ahrfl/fl mice used in the study. The starting weights of all the mice of the different genotypes, regardless of eventual diet type, were pooled. Statistical analysis was performed using a student t-test in GraphPad Prism. Error bars represent standard error of the mean.
(PDF)

S3 Fig Daily food intake comparisons between Creneg and Pdgfrα-Crepos Ahrfl/fl mice on control or HFD. Consumption of food of individually housed mice was measured over a week and average daily intake was calculated. Statistical analysis was performed using a One-Way ANOVA with multiple comparisons in GraphPad Prism. Error bars represent standard error of the mean.
(PDF)

S1 Table Assessment of steatosis in HFD mouse livers. ^a^Ten high-power fields (400X) centered on the terminal hepatic venule (for consistency) were scored for the percentage of the field with vacuolated cells. ^b^Rank was determined by degree of steatosis with a rank of 1 being highest. Ties were designated as equal numbers.
(PDF)

S1 Raw images (PDF)

## Abbreviations

AHR/Ahr: Aryl hydrocarbon receptor protein/gene
BAT: Brown adipose tissue
eWAT: Epididymal white adipose tissue (i.e. visceral white fat)
fl/fl: Flox/flox
GTT: Glucose tolerance test
HFD: High fat diet
ITT: Insulin tolerance test
iWAT: Inguinal white adipose tissue (i.e. subcutaneous white fat)
NE: Norepinephrine
PCB: Polychlorinated biphenyl
Pdgfrα: Platelet-derived growth factor receptor alpha
Q-RT-PCR: Quantitative reverse transcriptase polymerase chain reaction
TCDD: 2,3,7,8-Tetrachlorodibenzodioxin
UCP1/Ucp1: Uncoupling protein 1 protein/gene
WAT: White adipose tissue
