# Supplementary material for: Pdgfrα-Cre mediated knockout of the aryl hydrocarbon receptor protects mice from high-fat diet induced obesity and hepatic steatosis
Source: PLoS One. 2020 Jul 30;15(7):e0236741. doi: 10.1371/journal.pone.0236741 (PMC7392206; doi:10.1371/journal.pone.0236741)
Supplement: S1 Fig — A. Pdgfrα-Cre expression causes recombination to occur between the two loxP sites (black diamonds) surrounding exon 2 of Ahr (referred to as floxed), excising the exon and leaving one remaining loxP site. PCR using three primers (P1, P2, and P3) was performed to determine recombination (excision) status. In cells where no recombination occurs (upper structure), P2 and P3 amplify a 140 bp fragment (P1 and P3 are too far apart to achieve any appreciable amplification). In cells where recombination and excision occur (lower structure), the P2 site is removed and P1 and P3 are brought in close proximity to allow amplification of a 180 bp band. B. To verify AHR recombination, the indicated tissues were removed from Pdgfrα-Crepos Ahrfl/fl adult mice and processed for DNA. PCR was performed by using published primers [37]. The arrow indicates the upper 180 bp band that demonstrates recombination of the floxed Ahr gene (excised) in the tissue. The lower 140 bp band represents non-recombined (unexcised) Ahr. The pattern is typical of complex tissue such as adipose tissue where not all the different cell types express Pdgfrα-Cre. C. Stromal vascular fraction (SVF) consisting mainly of preadipocytes from BAT of 10 neonatal pups derived from the breeding of male Pdgfrα-Crepos/neg (heterozygous Cre)/Ahrfl/fl and female Ahrfl/fl mice that did not express Cre was isolated and cultured for one passage before DNA extraction and assessment for Cre positivity and Ahr recombination (excision) by PCR as described in A and in the Materials and Methods. Only the SVF that was Cre positive exhibited a pattern that verified excision. (PDF) [file pone.0236741.s001.pdf]

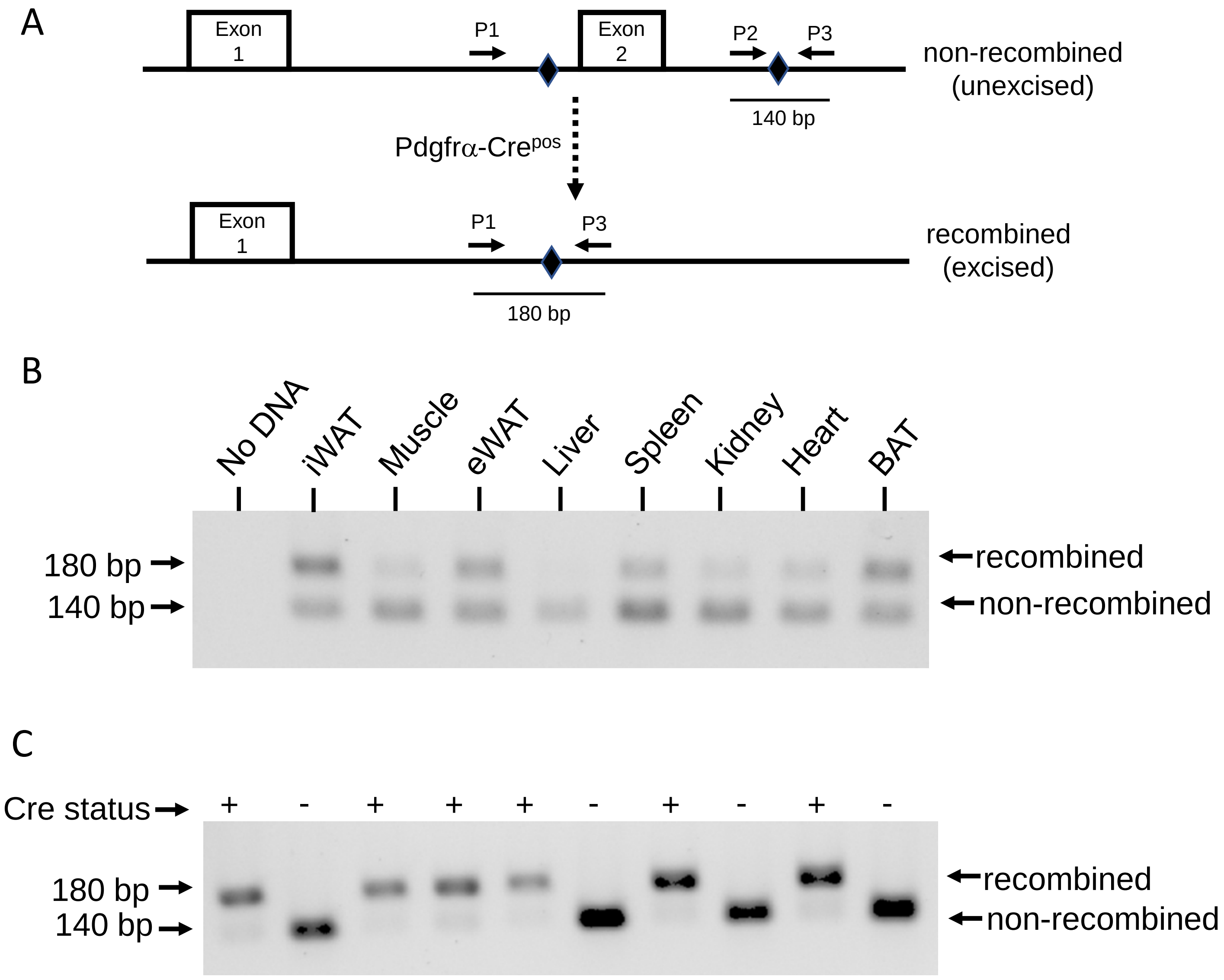

S1: Cre-mediated recombination of Ahr in different tissues and preadipocytes. **A**. Pdgfrα-Cre expression causes recombination to occur between the two loxP sites (black diamonds) surrounding exon 2 of Ahr (referred to as floxed), excising the exon and leaving one remaining lox site. PCR using three primers (P1, P2, and P3) was performed to determine recombination (excision) status. In cells where no recombination occurs (upper structure), P2 and P3 amplify a 140 bp fragment (P1 and P3 are too far apart to achieve any appreciable amplification). In cells where recombination and excision occur (lower structure), the P2 site is removed and P1 and P3 are brought in close proximity to allow amplification of a 180 bp band. **B**. To verify AHR recombination, the indicated tissues were removed from Pdgfrα-Cre^{pos} Ahr$^{fl/fl}$ adult mice and processed for DNA. PCR was performed by using published primers [37]. The arrow indicates the upper 180 bp band that demonstrates recombination of the floxed Ahr gene (excised) in the tissue. The lower 140 bp band represents non-recombined (unexcised) Ahr. The pattern is typical of complex tissue such as adipose tissue where not all the different cell types express Pdgfrα-Cre. **C**. Stromal vascular fraction (SVF) consisting mainly of preadipocytes from BAT of 10 neonatal pups derived from the breeding of male Pdgfrα-Cre+/- (heterozygous Cre)/Ahr$^{fl/fl}$ and female Ahr$^{fl/fl}$ mice that did not express Cre was isolated and cultured for one passage before DNA extraction and assessment for Cre positivity and Ahr recombination (excision) by PCR as described in A and in the Materials and Methods. Only the SVF that was Cre positive exhibited a pattern that verified excision.
