# Supplementary material for: Pdgfrα-Cre mediated knockout of the aryl hydrocarbon receptor protects mice from high-fat diet induced obesity and hepatic steatosis
Source: PLoS One. 2020 Jul 30;15(7):e0236741. doi: 10.1371/journal.pone.0236741 (PMC7392206; doi:10.1371/journal.pone.0236741)
Supplement: S2 Fig — The starting weights of all the mice of the different genotypes, regardless of eventual diet type, were pooled. Statistical analysis was performed using a student t-test in GraphPad Prism. Error bars represent standard error of the mean. (PDF) [file pone.0236741.s002.pdf]

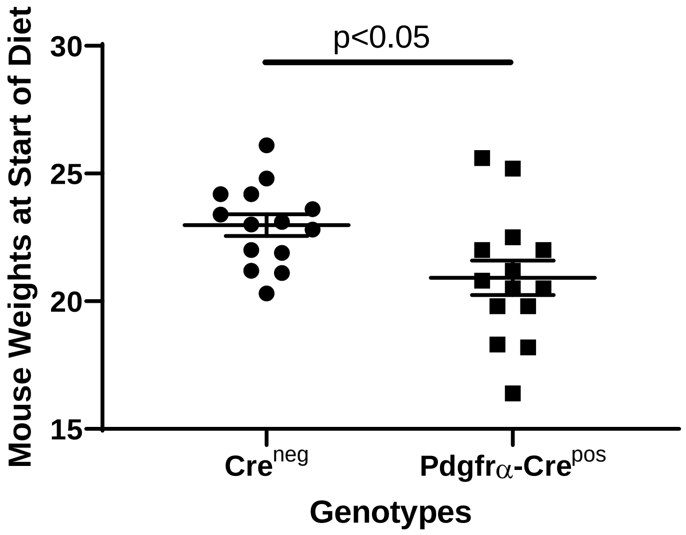

S2: Starting weights of 6- to 7-week old male Cre^{neg} or Pdgfrα-Cre^{pos} Ahr$^{fl/fl}$ mice used in the study. The starting weights of all the mice of the different genotypes, regardless of eventual diet type, were pooled. Statistical analysis was performed using a student t-test in GraphPad Prism. Error bars represent standard error of the mean.
