# Supplementary material for: Pdgfrα-Cre mediated knockout of the aryl hydrocarbon receptor protects mice from high-fat diet induced obesity and hepatic steatosis
Source: PLoS One. 2020 Jul 30;15(7):e0236741. doi: 10.1371/journal.pone.0236741 (PMC7392206; doi:10.1371/journal.pone.0236741)
Supplement: S3 Fig — Consumption of food of individually housed mice was measured over a week and average daily intake was calculated. Statistical analysis was performed using a One-Way ANOVA with multiple comparisons in GraphPad Prism. Error bars represent standard error of the mean. (PDF) [file pone.0236741.s003.pdf]

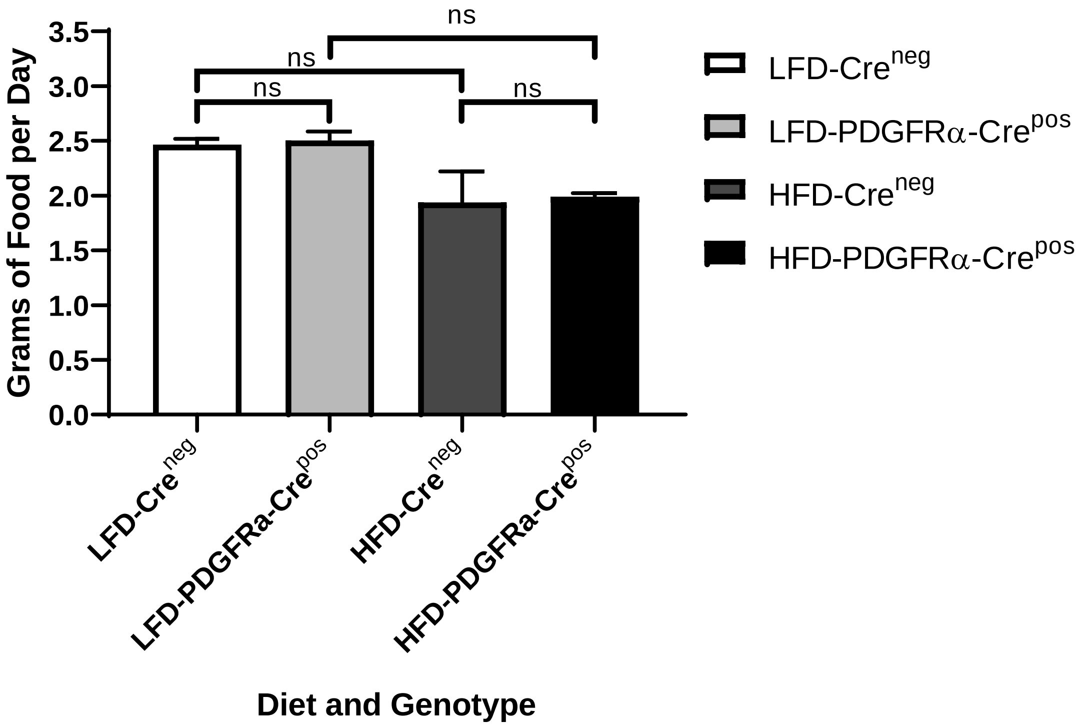

S3: Daily food intake comparisons between Cre^{neg} and Pdgfrα-Cre^{pos} Ahr$^{fl/fl}$ mice on control or HFDdiet. Consumption of food of individually housed mice was measured over a week and average daily intake was calculated. Statistical analysis was performed using a One-Way ANOVA with multiple comparisons in GraphPad Prism. Error bars represent standard error of the mean.

:
