# Supplementary material for: Pdgfrα-Cre mediated knockout of the aryl hydrocarbon receptor protects mice from high-fat diet induced obesity and hepatic steatosis
Source: PLoS One. 2020 Jul 30;15(7):e0236741. doi: 10.1371/journal.pone.0236741 (PMC7392206; doi:10.1371/journal.pone.0236741)
Supplement: S1 Table — aTen high-power fields (400X) centered on the terminal hepatic venule (for consistency) were scored for the percentage of the field with vacuolated cells. bRank was determined by degree of steatosis with a rank of 1 being highest. Ties were designated as equal numbers. (PDF) [file pone.0236741.s004.pdf]

**Supplemental Table I: Assessment of Steatosis in HFD Mouse Livers**

| Mouse number | [a]Field 1 | 2 | 3 | 4 | 5 | 6 | 7 | 8 | 9 | 10 | Steatosis Range | [b]Steatosis Rank |
|---|---|---|---|---|---|---|---|---|---|---|---|---|
| $Cre^{neg}$-1 | >75% | >75% | >75% | >50% | >50% | >50% | >75% | >75% | >75% | >75% | 50-75% | 3 |
| $Cre^{neg}$-2 | >75% | >75% | >75% | >75% | >75% | >75% | >75% | >75% | >75% | >75% | 75-100% | 1 |
| $Cre^{neg}$-3 | >75% | >75% | >75% | >75% | >75% | >75% | ~50% | >75% | >75% | >75% | 75% | 2 |
| $Cre^{neg}$-4 | ~15% | ~10% | ~10% | ~10% | ~20% | ~20% | ~25% | ~15% | ~15% | ~20% | 15-20% | 4 |
| | | | | | | | | | | | | |
| $Cre^{pos}$-1 | <5% | <5% | <5% | <5% | <5% | <5% | <5% | <5% | <5% | <5% | <5% | 5 |
| $Cre^{pos}$-2 | <5% | <5% | <5% | <5% | <5% | <5% | <5% | <5% | <5% | <5% | <5% | 5 |
| $Cre^{pos}$-3 | <5% | <5% | <5% | <5% | <5% | <5% | <5% | <5% | <5% | <5% | <5% | 5 |
| $Cre^{pos}$-4 | <5% | <5% | <5% | <5% | <5% | <5% | <5% | <5% | <5% | <5% | <5% | 5 |

[a]Ten high-power fields (400X) centered on the terminal hepatic venule (for consistency) were scored for the percentage of the field with vacuolated cells

[b]Rank was determined by degree of steatosis with a rank of 1 being highest. Ties were designated as equal numbers.
