# Supplementary figures and images for: Pdgfrα-Cre mediated knockout of the aryl hydrocarbon receptor protects mice from high-fat diet induced obesity and hepatic steatosis
Source: PLoS One. 2020 Jul 30;15(7):e0236741. doi: 10.1371/journal.pone.0236741 (PMC7392206; doi:10.1371/journal.pone.0236741)

Supplemental Figure 1B Raw Image

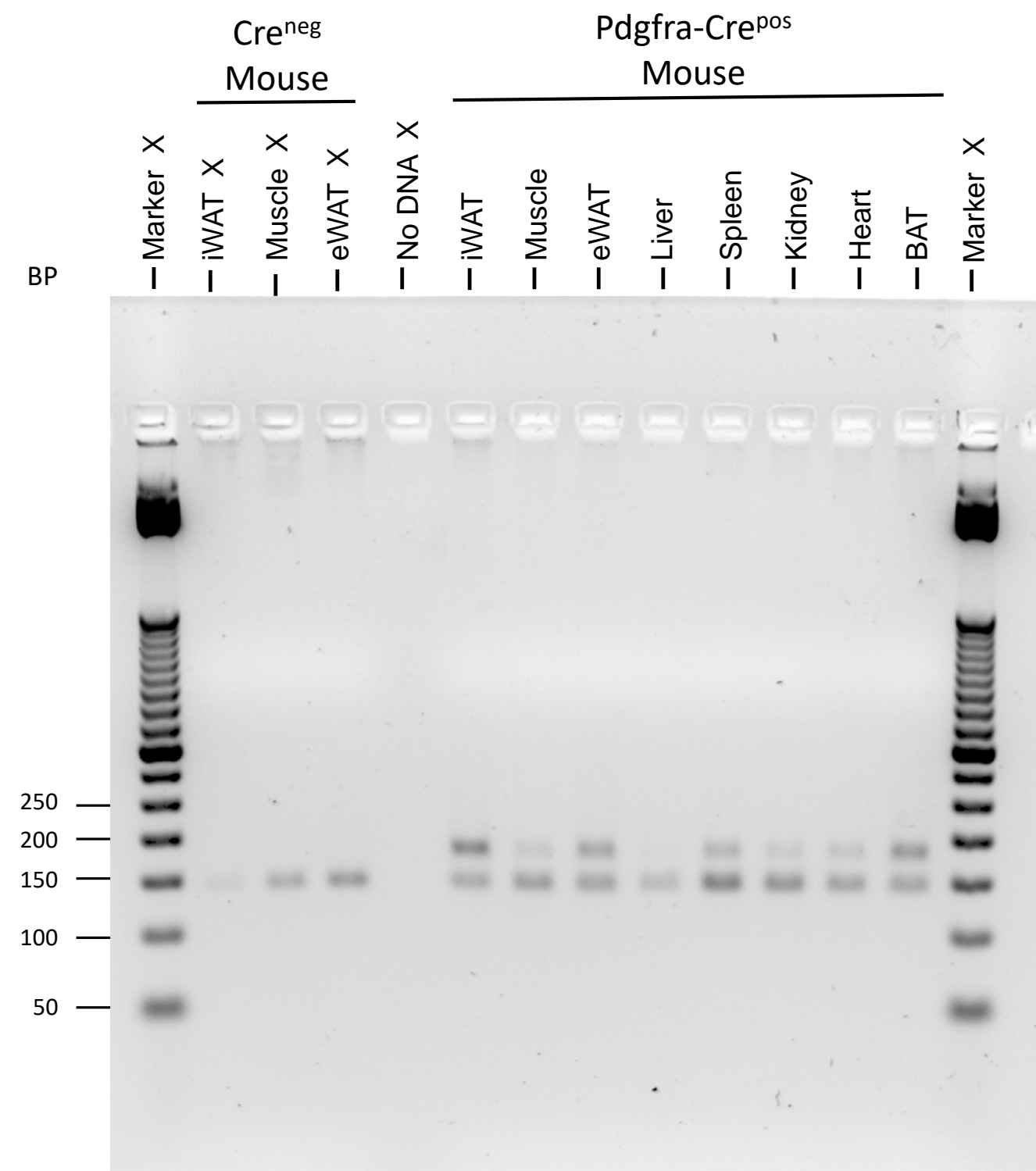

Supplemental Figure 1C Raw Image

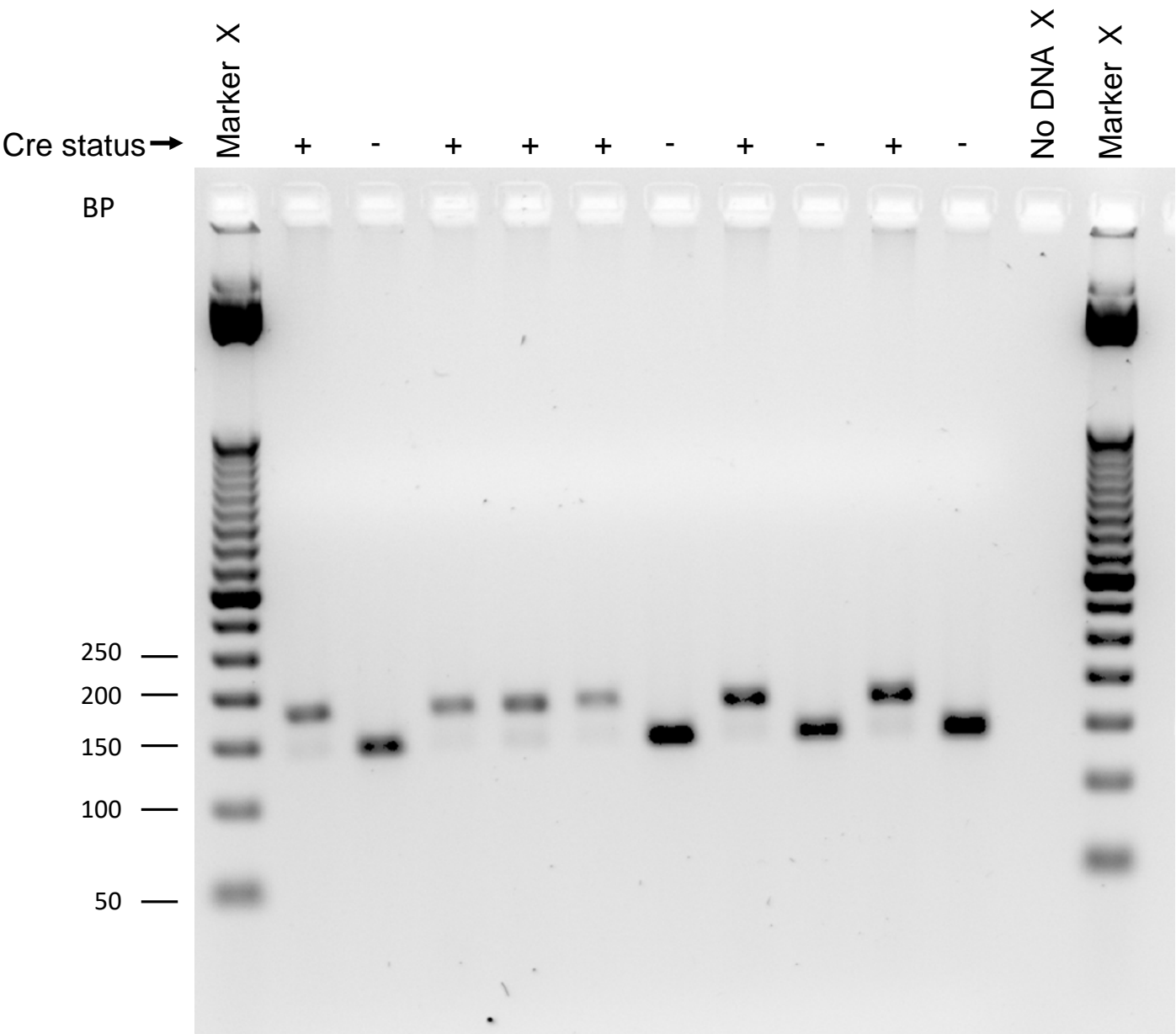

Supplement: S1 Raw images — (PDF) [file pone.0236741.s005.pdf]
